# Supplementary material for: Inflammatory Blood Signature Related to Common Psychological Comorbidity in Chronic Pain
Source: Biomedicines. 2023 Feb 27;11(3):713. doi: 10.3390/biomedicines11030713 (PMC10045222; doi:10.3390/biomedicines11030713)
Supplement: Supplementary file 1 [file biomedicines-11-00713-s001.zip › biomedicines-2181913-supplementary.pdf]

Inflammatory blood signature related to common psychological comorbidity in chronic pain

Bianka Karshikoff, Karin Wåhlén, Jenny Åström, Mats Lekander, Linda Holmström and Rikard K. Wicksell

Supplementary figures

The following score plots are based on the principal component analysis (PCA, Figure 2) including all proteins as x-variables. To get an overview of the clinical picture of each identified subgroups identified by the hierarchical cluster analysis (HCA), the score plots (showing the included observations or individual chronic pain patients) were sized and colored according to different clinical features. Each plot shows the first two principal components (t[1] and t[2]). The size of the geometrical shape represents higher self-reported score in the clinical variable of choice. The identified groups according to HCA were subgroup 1 = Squares, Subgroup 2 = circles, and subgroup 3 = triangles. Each individual score plot shows the distribution among the study population of sex (Figure S1), pain intensity (Figure S2), age (Figure S3), BMI (Figure S4), anxiety (GAD-9, Figure S5), and depression (PHQ-9, Figure S6).

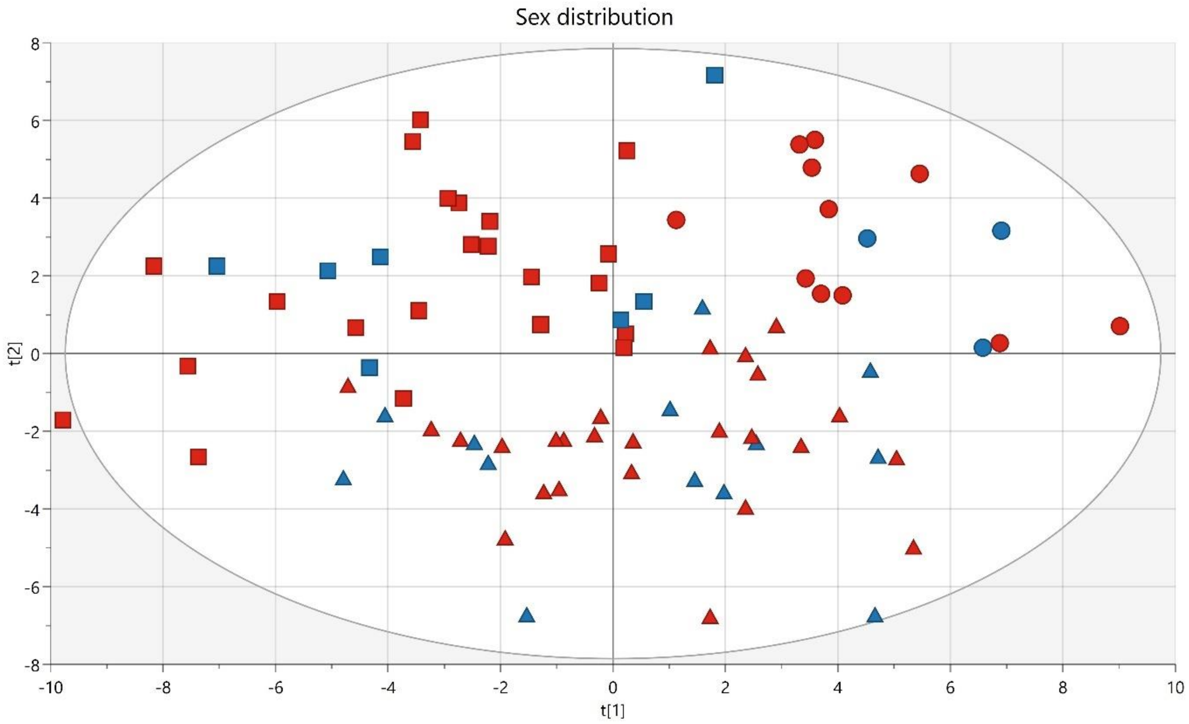

Figure S1. Score plot showing sex distribution among observations. Red = Women, blue = Men. Squares = group 1, circles = group 2, triangles = group 3.

Inflammatory blood signature related to common psychological comorbidity in chronic pain

Bianka Karshikoff, Karin Wåhlén, Jenny Åström, Mats Lekander, Linda Holmström and Rikard K. Wicksell

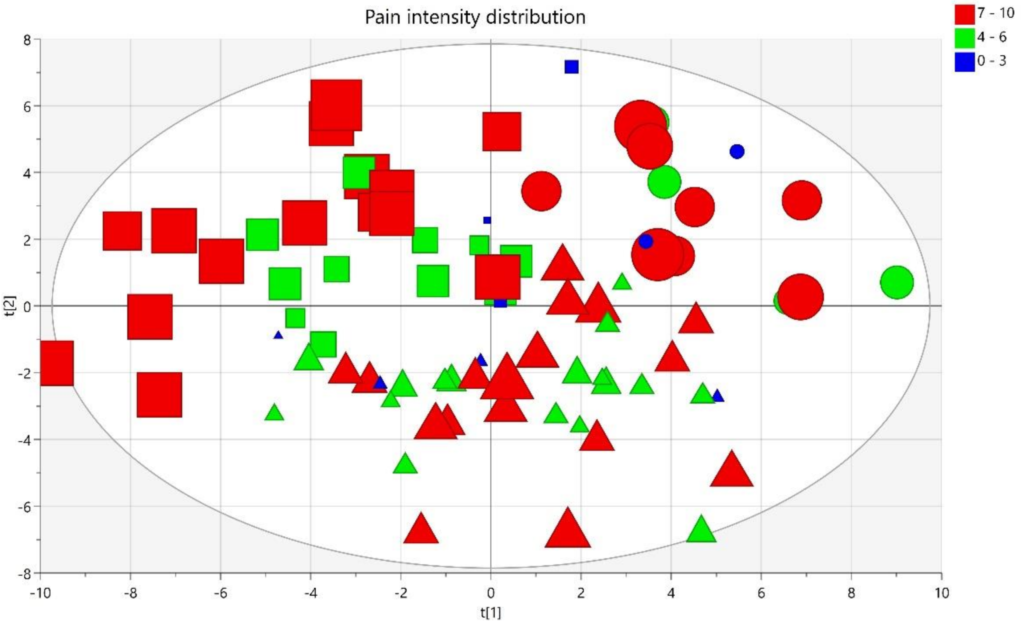

Figure S2. Pain intensity distribution among the three subgroups. The score plot show each observation and is colored according to self-reported pain intensity ranging from 0-10, where 0 = no pain, and 10 = worst imaginable pain. Blue = mild pain intensity (0-3), green = moderate pain intensity (4-6), and red = severe pain intensity (7-10). Each observation is also sized according to self-reported pain intensity, the larger size, the larger self-reported pain. Squares = group 1, circles = group 2, triangles = group 3**.**

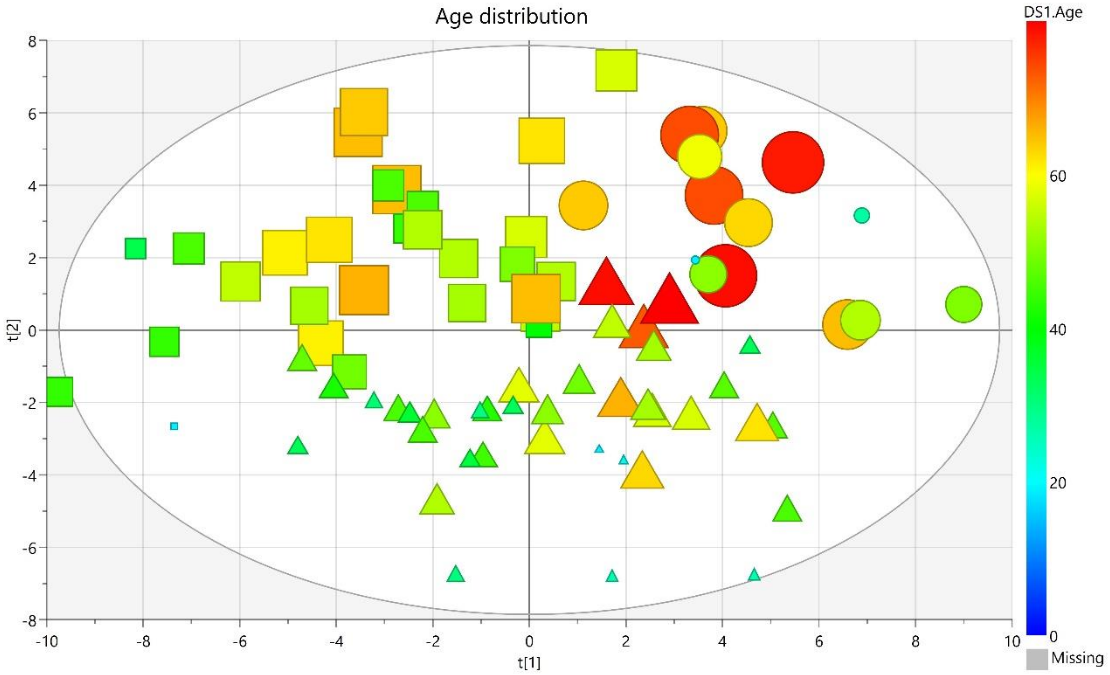

Figure S3. Score plot displaying the age distribution among each subgroup. The color bar to the right in the figure shows the age span (19-80 years). Each observation (squares = Group 1, circles = Group 2, or triangles = group 3) is sized according to age, the larger the size the higher age.

Inflammatory blood signature related to common psychological comorbidity in chronic pain

Bianka Karshikoff, Karin Wåhlén, Jenny Åström, Mats Lekander, Linda Holmström and Rikard K. Wicksell

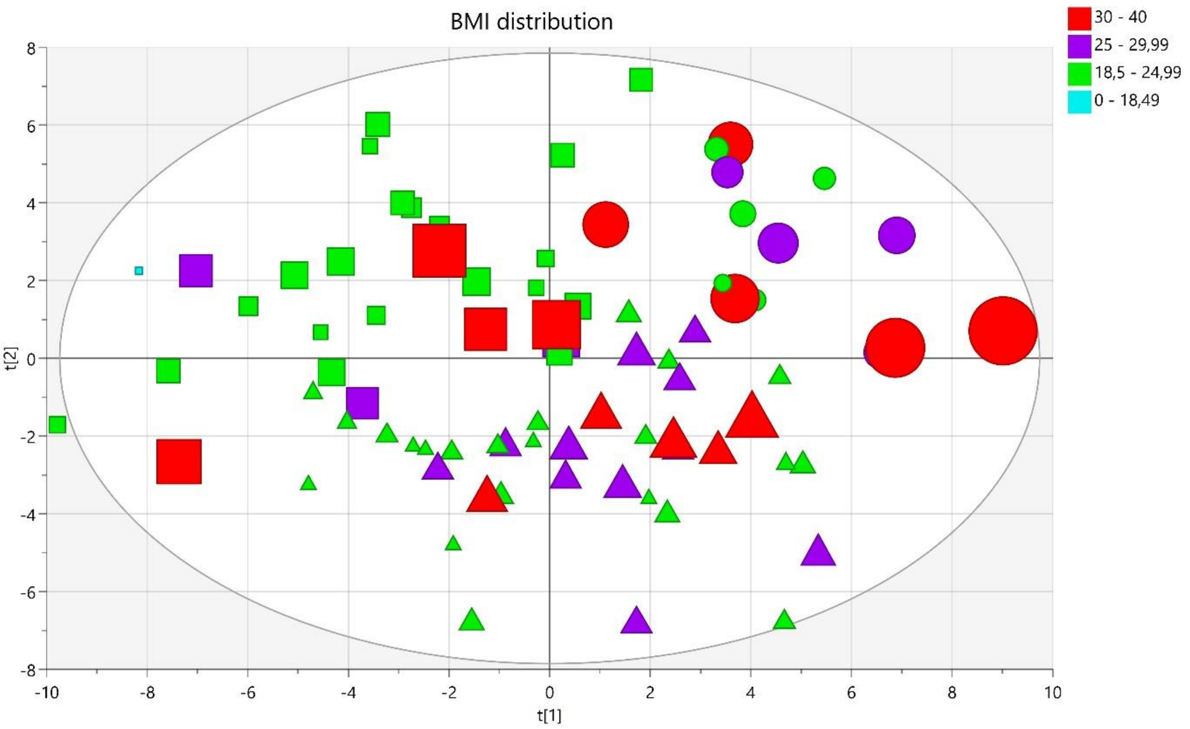

Figure S4. BMI distribution among the three subgroups. Each observation in the score plot is colored according to WHO standards of BMI: underweight <18.5, normal weight 18.5-24.99, overweight 25.0-29.99, obese >30. Each observation is sized according to BMI, the larger the size the higher BMI. Squares = group 1, circles = group 2, triangles = group 3.

Inflammatory blood signature related to common psychological comorbidity in chronic pain

Bianka Karshikoff, Karin Wåhlén, Jenny Åström, Mats Lekander, Linda Holmström and Rikard K. Wicksell

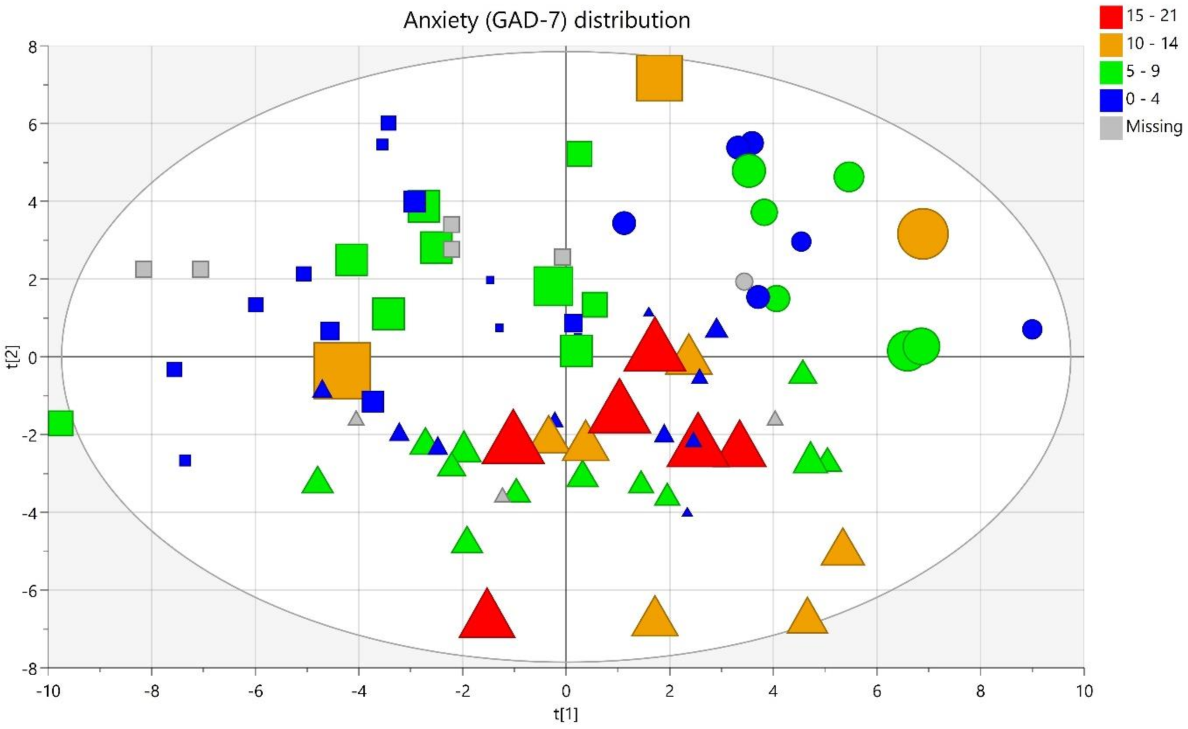

Figure S5. Distribution of anxiety based on self-reported GAD-7 questionnaire. The larger the geometrical shape the larger self-reported GAD-7 score. The cut-off scores for anxiety are: none 0-4 (blue), mild 5-9 (green), moderate 10-14 (yellow), severe 15-21 (red). Grey color indicates missing data in questionnaire. Squares = group 1, circles = group 2, triangles = group 3.

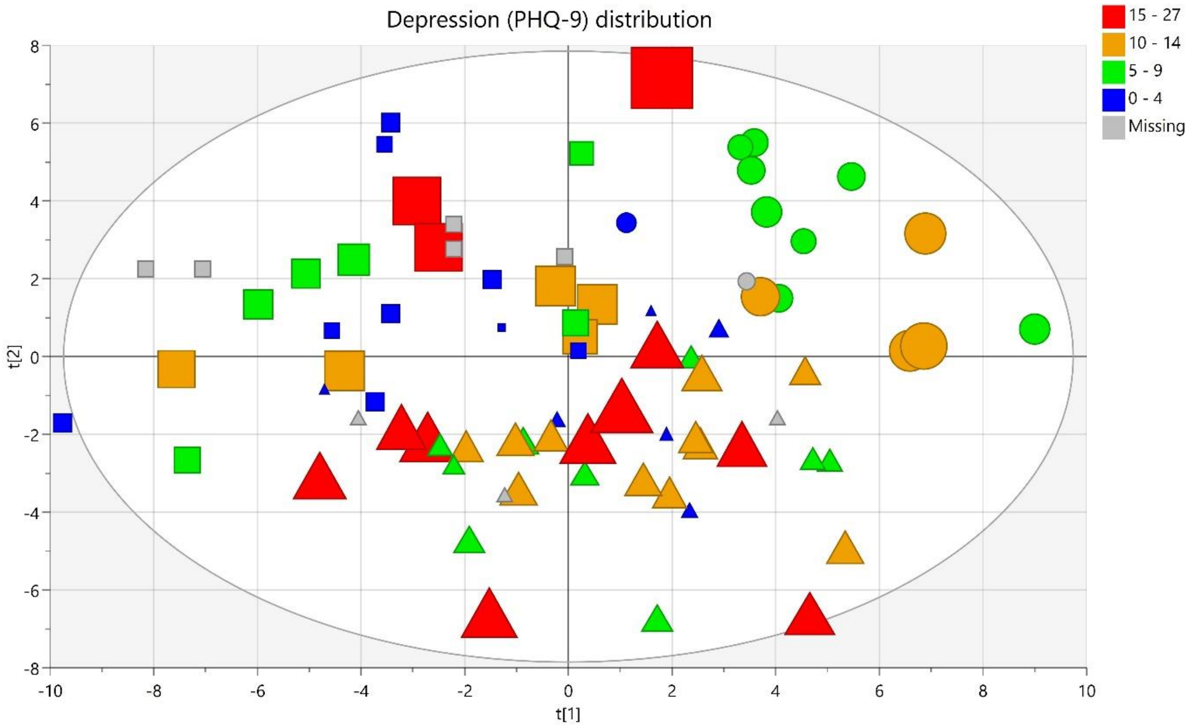

Figure S6. Distribution of depression based on self-reported PHQ-9 questionnaire. The larger the geometrical shape the larger self-reported PHQ-9 score. The cut-off scores for depression are: none 0-4 (blue), mild 5-9 (green), moderate 10-15 (yellow), moderate/severe 15-27 (red). Grey color indicates missing data in questionnaire. Squares = group 1, circles = group 2, triangles = group 3.

Inflammatory blood signature related to common psychological comorbidity in chronic pain

Bianka Karshikoff, Karin Wåhlén, Jenny Åström, Mats Lekander, Linda Holmström and Rikard K. Wicksell

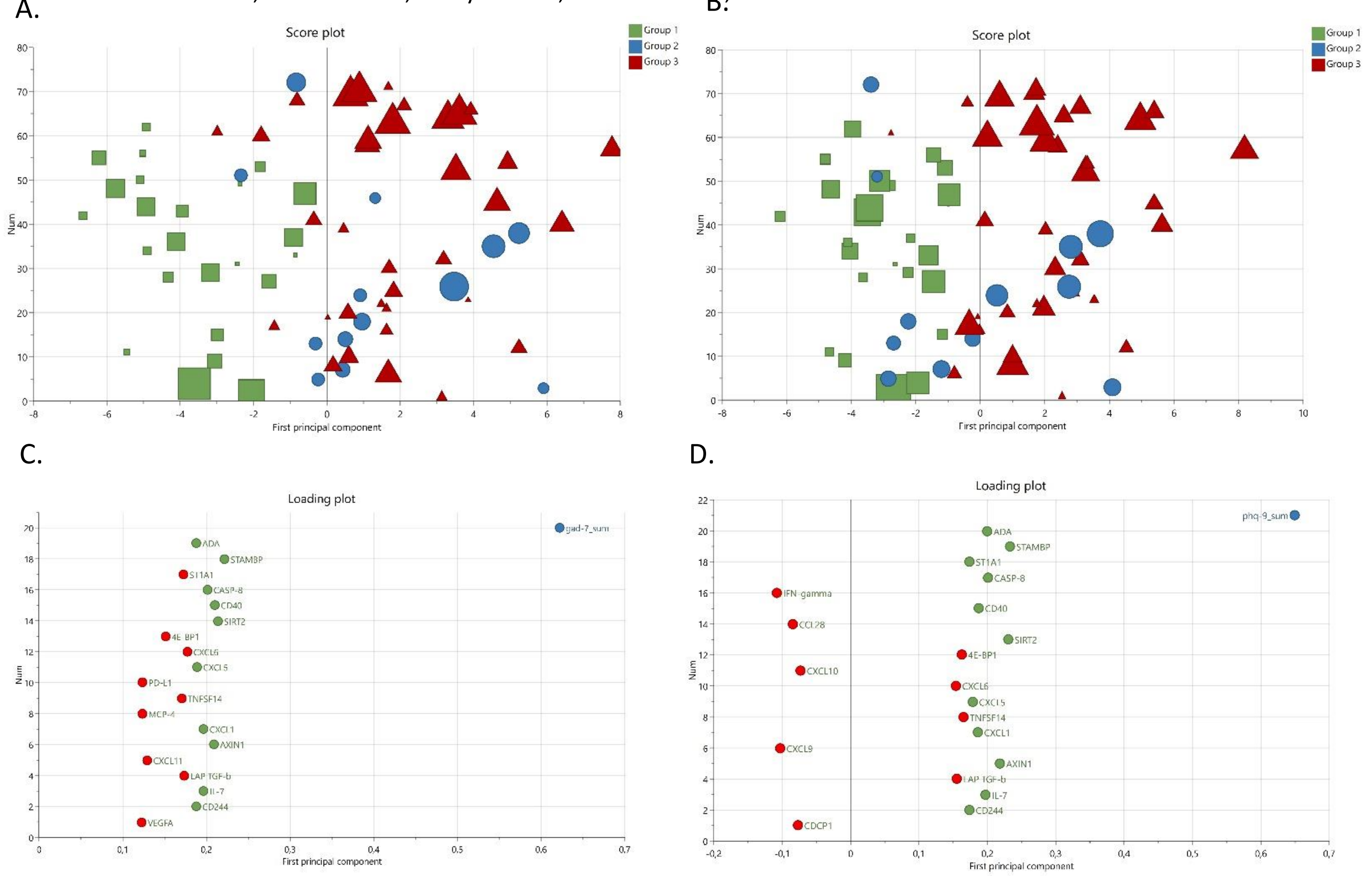

Figure S7. OPLS regression of anxiety (GAD-9) and depression (PHQ-9). In the score plots showing each observation for **A)** Anxiety (GAD-9) and **B)** Depression (PHQ-9), the geometrical shapes represent the three different subgroups identified by HCA in the PCA of protein. In the lower loading plots, **C)** Anxiety (GAD-7) and, **D)** Depression (PHQ-9), the green dots represent significant proteins with a VIP value>1.0. Red dots = non-significant proteins (VIP < 1.0). The larger the size of the geometrical shapes, the higher self-reportedPHQ-9 or GAD-9 scores. All significant proteins were associated with higher PHQ-9 and GAD-7 scores.

Bianka Karshikoff, Karin Wåhlén, Jenny Åström, Mats Lekander, Linda Holmström and Rikard K. Wicksell

Supplementary Tables

Table S1. Proteins intercorrelated to detected subgroups in PCA.

Protein name	Uniprot Protein Accession number	Loadings p[1]	Loadings p[2]
**Subgrupp 1 (squares)**			
**uPA**	P00749	0.007701	0.115283
**CST5**	P28325	0.014504	0.080477
**SCF**	P21583	-0.02967	-0.01695
**LIF-R**	P42702	-0.00129	0.137405
**TNF**	P01375	0.021701	0.010077
**DNER**	Q8NFT8	-0.05688	0.063711
**NT-3**	P20783	-0.01012	-0.00926
**TWEAK**	O43508	-0.03638	-0.00723
**TNFB**	P01374	0.008115	0.076902
**Cortisol**	-	-0.00779	0.033713
**Subgrupp 2 (circles)**			
**IL-8**	P10145	0.073215	0.087761
**VEGFA**	P15692	0.18589	0.015591
**MCP-3**	P80098	0.159124	0.028274
**GDNF**	P39905	0.10588	0.088696
**CDCP1**	Q9H5V8	0.092089	0.181317
**OPG**	O00300	0.101336	0.135738
**IL-6**	P05231	0.129856	0.06312
**MCP-1**	P13500	0.122117	0.168021
**IL-17A**	Q16552	0.06591	0.035902
**TRAIL**	P50591	0.092675	0.074463
**CXCL9**	Q07325	0.075454	0.212705
**CCL4**	P13236	0.124929	0.068135
**CD6**	P30203	0.014065	0.138969
**IL-18**	Q14116	0.095744	0.030416
**SLAMF1**	Q13291	0.08616	0.046251
**TGF-alpha**	P01135	0.131406	0.105285
**CCL11**	P51671	0.093557	0.175338
**FGF-23**	Q9GZV9	0.060616	0.052098
**FGF-21**	Q9NSA1	0.102735	0.075645
**CCL19**	Q99731	0.089788	0.134327
**IL-15RA**	Q13261	0.110022	0.160302
**IL-10RB**	Q08334	0.135783	0.112928
**IL-18R1**	Q13478	0.117285	0.048255
**PD-L1**	Q9NZQ7	0.162742	0.042569
**TRANCE**	O14788	0.076545	0.039912
**HGF**	P14210	0.183636	0.047935

**IL-12B**	P29460	0.076319	0.143504
**MMP-10**	P09238	0.031954	0.106529
**IL-10**	P22301	0.110218	0.058349
**CCL23**	P55773	0.030913	0.073926
**CD5**	P06127	0.039843	0.177607
**CCL3**	P10147	0.164579	0.10203
**Flt3L**	P49771	0.046692	0.154488
**CXCL10**	P02778	0.123533	0.174504
**CCL28**	Q9NRJ3	0.023178	0.139282
**EN-RAGE**	P80511	0.054851	0.063496
**IFN-gamma**	P01579	0.032722	0.133942
**FGF-19**	O95750	0.040216	0.103356
**CCL25**	O15444	0.066201	0.121163
**CX3CL1**	P78423	0.021749	0.143576
**TNFRSF9**	Q07011	0.110524	0.169327
**CCL20**	P78556	0.074937	0.079061
**CSF-1**	P09603	0.121774	0.152681
**hs_crp**	P02741	0.104404	0.027856
**Subgroup 3 (triangles)**			
**CD8A**	P01732	0.044268	0.014638
**CD244**	Q9BZW8	0.165331	-0.11223
**IL-7**	P13232	0.174802	-0.17432
**LAP TGF-beta-1**	P01137	0.1910	-0.05751
**CXCL11**	O14625	0.171861	-0.04536
**AXIN1**	O15169	0.163262	-0.21499
**OSM**	P13725	0.126696	-0.03647
**CXCL1**	P09341	0.175852	-0.1556
**MCP-4**	Q99616	0.171629	-0.01139
**TNFSF14**	O43557	0.187033	-0.08975
**IL-10RA**	Q13651	0.061916	0.006705
**MMP-1**	P03956	0.084347	0.000789
**CXCL5**	P42830	0.170783	-0.16153
**CXCL6**	P80162	0.183431	-0.08609
**4E-BP1**	Q13541	0.12427	-0.12448
**SIRT2**	Q8IXJ6	0.157645	-0.22338
**CD40**	P25942	0.205989	-0.12672
**MCP-2**	P80075	0.109099	-0.02847
**CASP-8**	Q14790	0.179056	-0.14337
**ST1A1**	P50225	0.119242	-0.1716
**STAMBP**	O95630	0.168374	-0.22128
**ADA**	P00813	0.144885	-0.17022
**sr_ESR**	-	0.090901	0.002094

Table S2. Background and clinical data subgroups

Variable	Subgroup 1 (n=29)	Subgroup 2 (n=14)	Subgroup 3 (n=38)	Kruskal-Wallis test		Subgroup 1 vs 2	Subgroup 1 vs 3	Subgroup 2 vs 3
	**Mean (SD)**			**H**	***P-value***	**Adj. *P-value***		
**Background data**								
Age	52.59 (10.61)	58.64 (17.77)	46.89 (15.04)	8.632	**0.0134**	0.4507	0.2703	**0.0138**
BMI	24.19 (4.03)	27.89 (5.70)	25.22 (4.07)	4.27	0.1184	0.1167	1.00	0.4642
Sex, Females/ Males % (n)	75.7 % (22) / 24.1% (7)	78.6% (11)/ 21.4% (3)	65.8 % (25)/ 34.2 % (13)	-	-	-	-	-
**Pain characteristics**								
Pain area number	4.65 (2.97)	4.01 (2.97)	4.92 (3.04)	0.4814	0.7861	1.00	1.00	1.00
Pain intensity	6.38 (1.86)	6.50 (1.871)	6.026 (1.91)	1.243	0.5373	1.00	1.00	1.00
Pain duration (years)	10.43 (6.29)	15.73 (16.02)	10.41 (6.53)	0.424	0.8090	1.00	1.00	1.00
**Psychological comorbidity**								
Depression (PHQ-9)	7.667 (4.896)	8.385 (2.931)	10.86 (5.996)	4.532	0.1037	1.00	0.1114	0.8132
Anxiety (GAD-7)	4.50 (3.612)	5.692 (2.658)	7.857 (5.151)	6.907	**0.0316**	0.8307	**0.0260**	0.9677
**Other clinical parameters**								
General health (SRH-5)	2. 86 (0.915)	2.93 (0.99)	2.95 (1.06)	0.1256	0.9392	1.00	1.00	1.00
Insomnia (ISI)	13.56 (6.68)	13.57 (6.63)	13.11 (6.71)	0.0146	0.9927	1.00	1.00	1.00
Stress (PSS)	22.0 (9.63)	15.0 (5.88)	20.44 (9.32)	4.425	0.1092	0.1278	1.0	0.2106
Physical wellbeing (PCS-12)	32.5 (6.97)	32.97 (12.2)	33.23 (8.19)	0.0147	0.9927	1.00	1.00	1.00
Mental wellbeing (MCS-12)	37.2 (13.35)	49.28 (11.17)	38.32 (12.83)	6.634	**0.0363**	*0.0570*	1.00	***0.0502***

Table S3. Subgroup comparison of significant proteins from OPLS models of depression and anxiety.

Protein name	**Uniprot Protein Accession number**	Subgroup 1 (n=29)	Subgroup 2 (n=14)	Subgroup 3 (n=38)	Kruskal-Wallis test		Subgroup 1 vs 2	Subgroup 1 vs 3	Subgroup 2 vs 3
	**NPX Mean (SD)**				**H**	***P-value***	**Adj. *P-value***		
**CD244**	Q9BZW8	6.430 (0.299)	6.790 (0.377)	6.860 (0.284)	25.90	**<0.0001**	**0.0055**	**<0.0001**	1.00
**IL-7**	P13232	2.214 (0.395)	3.056 (0.490)	3.209 (0.531)	44.40	**<0.0001**	**<0.0001**	**<0.0001**	1.00
**AXIN1**	O15169	4.338 (0.799)	5.536 (0.612)	6.179 (0.698)	49.66	**<0.0001**	**0.0049**	**<0.0001**	0.0685
**CXCL1**	P09341	8.012 (0.728)	9.150 (0.500)	9.244 (0.612)	35.76	**<0.0001**	**0.0003**	**<0.0001**	1.00
**CXCL5**	P42830	9.648 (1.179)	11.32 (0.647)	11.65 (0.771)	37.84	**<0.0001**	**0.0009**	**<0.0001**	0.9405
**SIRT2**	Q8IXJ6	4.707 (0.746)	5.948 (0.808)	6.694 (0.627)	51.55	**<0.0001**	**0.0079**	**<0.0001**	**0.0339**
**CD40**	P25942	10.91 (0.314)	11.60 (0.299)	11.57 (0.317)	40.95	**<0.0001**	**<0.0001**	**<0.0001**	1.00
**Caspase-8**	Q14790	1.026 (0.252)	1.566 (0.493)	1.606 (0.326)	39.66	**<0.0001**	**0.0007**	**<0.0001**	0.8521
**ST1A1**	P50225	2.295 (0.709)	2.975 (0.627)	3.381 (0.579)	31.02	**<0.0001**	**0.0303**	**<0.0001**	0.2606
**STAMBP**	O95630	5.385 (0.553)	6.473 (0.772)	7.029 (0.620)	51.74	**<0.0001**	**0.0019**	**<0.0001**	0.1041
**ADA**	P00813	4.832 (0.262)	5.435 (0.617)	5.490 (0.417)	39.17	**<0.0001**	0.0004	**<0.0001**	1.00

Bianka Karshikoff, Karin Wåhlén, Jenny Åström, Mats Lekander, Linda Holmström and Rikard K. Wicksell

Table S4. Significant proteins for the OPLS models investigating correlation with age. Proteins are sorted according to highest VIP-value. Bold marked proteins are proteins that were found significant in the OPLS-DA modelcomparing patients with moderate/high or low /no psychological comorbidity. # = protein found in the OPLS models of depression (PHQ-9) and anxiety (GAD-7).

Age

Protein name	**Uniprot Protein Accession number**	**VIPpred**	**p(corr)**
CXCL9	Q07325	2.88046	0.778189
CXCL10	P02778	2.63727	0.712466
CDCP1	Q9H5V8	2.1949	0.592938
CCL28	Q9NRJ3	2.08892	0.564361
OPG	O00300	2.0123	0.543597
CCL11	P51671	1.92525	0.520187
Flt3L	P49771	1.87183	0.505567
CST5	P28325	1.64666	0.444777
FGF-21	Q9NSA1	1.4996	0.405091
MCP-4	Q99616	1.48922	0.402464
MCP-1	P13500	1.43749	0.388364
IL-12B	P29460	1.28246	0.346422
CCL3	P10147	1.27309	0.344007
TNF	P01375	1.14689	-0.30985
MCP-3	P80098	1.14239	0.308739
CCL25	O15444	1.07536	0.290446
HGF	P14210	1.0654	0.287859
CXCL11	O14625	1.05043	0.283842
***ADA*#**	P00813	1.0382	-0.28034
SCF	P21583	1.0343	-0.27939
IL-6	P05231	1.01946	0.275388
IL-15RA	Q13261	1.00705	0.271993
